# Intraglomerular Inflammation as a Guide for Mycophenolate Mofetil-Based Treatment in IgA Nephropathy

**DOI:** 10.3390/diagnostics15162101

**Published:** 2025-08-20

**Authors:** Christodoulos Keskinis, Panagiotis Pateinakis, Maria Stangou

**Affiliations:** 1 School of Medicine, Aristotle University of Thessaloniki (AUTH), 54124 Thessaloniki, Greece; mstangou@auth.gr; 2 Department of Nephrology, Papageorgiou General Hospital, 56429 Thessaloniki, Greece; pateinakis@hotmail.com; 3 1st Department of Nephrology, Hippokration Hospital, School of Medicine, Aristotle University of Thessaloniki, 54642 Thessaloniki, Greece

**Keywords:** IgA nephropathy, mycophenolate mofetil, corticosteroids, intraglomerular inflammation, crescent, endocapillary hypercellularity

## Abstract

IgA nephropathy (IgAN) is the most prevalent primary glomerulonephritis worldwide, with a heterogeneous clinical course that may progress to end-stage kidney disease (ESKD) in approximately 20% of patients. Despite recent advances, including the U.S. Food and Drug Administration (FDA) approval of three novel agents, optimal therapeutic strategies remain uncertain, and access to new drugs is often limited. This underscores the need to evaluate established and widely available options such as mycophenolate mofetil (MMF). The aim of this review is to critically assess the role of MMF, either as monotherapy or in combination with systemic corticosteroids, in the treatment of IgAN based on evidence cited in the KDIGO 2024 Draft Guidelines. We analyzed seven major clinical studies—five randomized controlled trials and two long-term observational studies—with particular focus on the influence of histological activity on treatment outcomes. The Oxford classification was applied to explore whether specific histological variables correlate with prognosis and predict treatment response. Trials conducted in Chinese cohorts demonstrated significant benefits of MMF, including proteinuria reduction, delayed progression to ESKD, and improved long-term renal outcomes, particularly in patients with recent disease onset and active proliferative lesions such as endocapillary hypercellularity and crescent formation. In contrast, studies from Western populations generally failed to demonstrate comparable benefit possibly due to differences in disease chronicity, histopathological patterns, and genetic background. Overall, MMF appears most effective when initiated early and in patients with histologic evidence of intraglomerular inflammation. It may represent a viable steroid-sparing option in appropriately selected patients, particularly where access to newly approved agents is restricted. These population- and pathology-based differences highlight the need for individualized treatment decisions and further research to refine the therapeutic role of MMF in IgAN.

## 1. Introduction

IgAN, characterized by mesangial deposition of IgA-containing immune complexes, is the most common primary glomerulonephritis worldwide [1,2,3,4,5,6]. The clinical spectrum is highly heterogeneous, ranging from asymptomatic microscopic hematuria to rapidly progressive glomerulonephritis leading to ESKD [7,8]. Despite advancements in understanding the pathophysiology of IgAN, reliable biomarkers for diagnosis or prognosis remain limited [9,10,11,12]. Proteinuria and estimated glomerular filtration rate (eGFR) are widely used prognostic indicators and they are supported by Kidney Disease: Improving Global Outcomes (KDIGO) 2021 guidelines; however, these parameters are often influenced by comorbidities such as hypertension and diabetes mellitus and may not directly reflect IgAN-specific disease activity [10,13]. The Oxford classification system/MEST-C score, initially introduced in 2009 and revised in 2016, incorporates five histopathological variables: mesangial hypercellularity (M), endocapillary hypercellularity (E), segmental glomerulosclerosis (S), tubular atrophy/interstitial fibrosis (T), and cellular/fibrocellular crescents (C) [14,15,16,17,18]. While it is a validated prognostic tool in IgA nephropathy, it is not intended to guide therapeutic decision-making [15].

Currently, kidney biopsy remains the gold standard for diagnosis, as per the 2021 KDIGO guidelines [2,10]. Even galactose-deficient IgA1 (gd-IgA1), while central to the pathogenesis, has not been validated as a diagnostic or prognostic biomarker in clinical practice [19,20,21,22,23,24]. IgAN predominantly affects young adult males, and even in cases initially presenting with low-grade proteinuria (<1 g/day), long-term outcomes can be unfavorable [1,5]. Cohort studies have shown that seemingly mild disease may progress insidiously, culminating in ESKD over a period of 20–25 years. Recent data suggest revising the proteinuria threshold to <300 mg/day, as patients with persistent proteinuria above this limit, even with otherwise stable renal parameters, remain at considerable risk for disease progression [25].

Standard care includes optimized supportive therapy, comprising blood pressure control, renin–angiotensin system (RAS) blockade, and sodium-glucose cotransporter-2 inhibitors (SGLT2is) [26,27,28,29]. Nonetheless, a significant subset of patients with persistent proteinuria and histologically active lesions fail to achieve disease remission with supportive care alone. This has led to ongoing investigation of immunosuppressive strategies, primarily systemic corticosteroids and mycophenolate mofetil (MMF) [10,30,31,32].

Given the relatively young age of most IgAN patients and the chronic course of the disease, those who progress to ESKD often require dialysis for decades [1,33]. As such, achieving disease control early is essential [33]. Historically, systemic corticosteroids have been the cornerstone of immunosuppressive therapy in IgAN, particularly since the mid-1980s, when initial trials suggested significant efficacy [2,34,35,36]. However, the early studies supporting steroid use were limited by several key methodological flaws: suboptimal use of background antiproteinuric therapy, a lack of relapse data, and inadequate documentation of adverse events [34,35,36].

More recent high-quality trials, including the STOP-IgAN and TESTING studies, have reaffirmed the efficacy of corticosteroids in reducing proteinuria and delaying renal decline but have simultaneously raised serious concerns regarding safety [31,37,38]. Life-threatening infections and metabolic complications have limited the enthusiasm for high-dose, prolonged steroid use [30]. KDIGO 2021 guidelines now recommend systemic corticosteroids only under specific conditions and for a limited duration (typically 6 months), with a starting daily dose of 32 mg in most cases [29]. Emerging therapies, such as targeted-release budesonide (TRB), represent steroid-sparing alternatives and have shown promise in early-phase trials [2,39,40,41]. TRB, the first FDA-approved agent for IgAN, acts on mucosal immune responses in the gut to reduce gd-IgA1 production but is minimally absorbed (∼10%) systemically, thereby limiting its direct renal anti-inflammatory impact [2,42].

This context raises a critical question: What is the role of MMF in IgAN therapy? MMF was initially considered a glucocorticoid-sparing agent but is increasingly being explored as a primary immunosuppressive option, particularly in patients with evidence of intraglomerular inflammation [32]. Although several novel agents are under investigation, access remains limited, and cost may be prohibitive [43,44]. Even if approved, many of these therapies are expected to be expensive, often requiring repeated courses costing tens or hundreds of thousands of dollars per patient [2,13]. In this setting, MMF represents a more accessible and cost-effective alternative.

The recent KDIGO 2024 Draft includes revised considerations for MMF use, informed by new randomized controlled trials (RCTs) and observational data. MMF, an inhibitor of inosine monophosphate dehydrogenase, selectively impairs T and B lymphocyte proliferation and thus attenuates both antibody production and downstream inflammatory signaling [45]. Therefore, the agent appears to act relatively early in the immunologic cascade. As such, it is of particular interest to determine whether patients with active immunologic inflammation at the glomerular level respond to therapy, given that the agent does not exert its primary effect directly within the glomerulus.

## 2. Materials and Methods

This article represents a narrative review and incorporates studies referenced in the KDIGO 2024 Draft that have evaluated the use of MMF in IgAN patients. The goal of this review is to critically interpret the existing evidence that forms the basis of current guideline recommendations, rather than providing an exhaustive summary of the broad literature on MMF or IgAN treatment. Therefore, data from seven key clinical trials, five RCTs and two long-term observational studies, are included, focusing particularly on the predictive value of intraglomerular inflammation in guiding MMF-based immunosuppressive therapy [46,47,48,49,50,51,52]. The objective is to critically appraise, compare, and contextualize the findings of these studies. Despite the recent proliferation of clinical trials investigating novel therapeutic agents for IgAN, research into the role of MMF continues to be conducted, reflecting ongoing interest in its potential utility. According to current KDIGO guidelines, MMF is recommended primarily for Chinese populations, as available evidence does not support a consistent benefit in other ethnic groups [10]. Nevertheless, experienced nephrologists in various regions continue to advocate MMF as a reliable therapeutic option based on long-standing clinical experience and individualized patient responses.

## 3. Results

### 3.1. Timeline of Main Clinical Trials in MMF

Notably, the earliest studies, such as those by Tang et al. (2005, 2010), were conducted in Chinese populations and formed the basis for the initial optimism surrounding MMF’s potential benefits, particularly in East Asian cohorts [46,50]. Conversely, subsequent randomized controlled trials conducted in North America and Europe (e.g., Hogg et al., Frisch et al., Maes et al.) did not replicate these findings, potentially due to differences in population characteristics, histologic activity, or disease chronicity [48,49,52]. The more recent, large-scale Chinese studies by Hou et al. (2017, 2023) marked a significant advance in both methodological rigor and patient stratification [47,51]. These trials incorporated longer treatment durations, larger sample sizes, and histologic inclusion criteria, providing more robust evidence supporting MMF’s role in selected patient populations.

Table 1 summarizes the key structural and design elements of the studies evaluating the efficacy of MMF in the treatment of IgAN. These trials differ considerably in terms of geographic location, sample size, design type, intervention arms, and duration, reflecting the evolving and often region-specific nature of research on immunosuppression in IgAN.

These studies represent a cross-section of geographic and ethnic populations, allowing for comparison of MMF’s efficacy across diverse IgAN phenotypes. The earliest among these, the RCT by Tang et al. (2005) in China, enrolled 40 patients and compared MMF with conventional supportive care over a 24-week period, followed by 72 weeks of observational follow-up [50]. The study provided preliminary evidence that MMF may reduce proteinuria and preserve renal function. However, the restricted sample size and single-center recruitment limited the generalizability of these results [50]. Furthermore, the brief treatment duration contrasts with the typically prolonged course of IgAN, which may compromise conclusions regarding long-term efficacy [50].

Tang et al. (2010) presented a six-year observational follow-up of the same patient cohort [46]. Although not a randomized study, these long-term data provided valuable insights into the potential durability of MMF’s therapeutic effects [46]. The extended observational period, rare in IgAN research, is a major strength. However, the absence of a control group, potential loss to follow-up, and changes in concurrent therapies over time introduce confounding variables, weakening causal inferences regarding MMF’s efficacy [46].

In contrast, Hogg et al. (2015) conducted a multicenter, placebo-controlled RCT in the United States and Canada, enrolling 52 patients randomized to receive MMF or placebo for 12 months [52]. The rigorous methodology and Western population context offer an important counterbalance to Asian studies. Nevertheless, the trial found no statistically significant difference in proteinuria or renal function between groups. This discrepancy raises important questions about interethnic variability in treatment response and disease phenotype. Differences in the prevalence of active histologic lesions, such as endocapillary hypercellularity or crescents, may partly explain the lack of observed benefit in this cohort.

Frisch et al. also investigated MMF in a United States (USA)-based RCT with 32 patients treated over 24 months [49]. Despite the relatively long treatment period, the study reported no significant improvement in renal outcomes. The authors noted that many participants lacked active histologic inflammation, suggesting that the inclusion of patients with more indolent disease may have weakened any therapeutic signal [49]. This observation underscores the growing recognition of histologic stratification as a determinant of immunosuppressive responsiveness in IgAN [49].

Similarly, in Belgium, Maes et al. conducted one of the longest studies among MMF research, a 36-month RCT involving 34 IgAN patients [48]. While the extended timeframe is valuable, the study did not demonstrate significant clinical benefit [48]. As with other Western trials, limited statistical power and potential differences in disease phenotype may have influenced the neutral outcome.

Hou et al. (2017) further explored MMF’s utility by comparing MMF combined with low-dose corticosteroids with full-dose corticosteroids in a 176-patient RCT [47]. The study aimed to reduce corticosteroid exposure, a key concern given the adverse effects associated with long-term steroid treatment [47]. It showed that the MMF-plus-low-steroid arm achieved comparable efficacy in reducing proteinuria and preserving renal function, but with a superior safety profile [47]. This steroid-sparing strategy is of particular interest in clinical practice, especially for patients with contraindications to systemic corticosteroids [47].

A major advancement came from a large-scale Chinese RCT by Hou et al. [51] enrolling 170 patients randomized to receive either MMF plus supportive care or supportive care alone over 36 months [51].

This trial provided the most robust evidence to date supporting MMF’s efficacy, demonstrating significant reductions in proteinuria and the preservation of eGFR. Importantly, patients were stratified using the Oxford MEST-C classification, and the benefits were particularly pronounced in those with active lesions (E1, C1/2). The study’s rigorous design, adequate sample size, and histologic stratification enhance both its internal and external validity, at least within East Asian populations. Whether these findings can be generalized to Western populations remains uncertain, given the lack of similar high-quality data in non-Asian cohorts.

### 3.2. MMF Dosage and Co-Administered Treatment

The dosing regimens of mycophenolate mofetil (MMF), along with conservative treatments including renin–angiotensin system (RAS) blockade, represent critical variables in evaluating the efficacy and comparability of clinical trials in IgAN. Table 2 outlines the MMF dosing strategies and the uniform application of RAS inhibition across seven pivotal studies.

MMF dosing ranged from fixed regimens to weight-based approaches. In Tang et al. (2005), MMF was administered at 1.5–2 g/day, consistent with immunosuppressive practices in lupus nephritis and transplantation [50]. This was continued in their 2010 observational follow-up, though without detailed dosing restatement [46]. Importantly, both studies employed MMF as monotherapy alongside RAS blockade, allowing for relatively isolated evaluation of its effects. Hogg et al. (2015) utilized a weight-based dose of 25–36 mg/kg/day, approximately equivalent to 1.5–2 g/day in adults [52]. Patients received ACE inhibitor or ARB therapy and omega-3 fatty acids.

In Frisch et al., MMF was dosed at 1 g twice daily, while Maes et al. used 2 g/day in combination with sodium restriction and ACE inhibition [48,49]. Both trials excluded other immunosuppressants, offering cleaner assessments of MMF monotherapy, though the absence of histologic stratification may have obscured efficacy in subgroups with active disease.

Hou et al. (2023) adopted a tapering strategy, initiating MMF at 1.5 g/day and gradually reducing to 0.75–1 g/day over the treatment course [51]. All patients received losartan as RAS blockade. This structured approach likely enhanced tolerability while maintaining efficacy, contributing to the trial’s favorable outcomes. Earlier, Hou et al. (2017) administered MMF at 1.5 g/day in combination with low-dose corticosteroids, introducing a steroid-sparing protocol [47]. This regimen preserved immunosuppressive efficacy while reducing corticosteroid-related toxicity, offering a pragmatic balance in long-term IgAN care [47].

Across all trials, RAS blockade was uniformly applied, typically via ACE inhibitors or ARBs. This consistency offers a stable background upon which MMF’s additive value could be assessed. However, differences in MMF dose intensity, steroid use, and supportive interventions likely influenced the magnitude and durability of clinical responses. In summary, while RAS blockade serves as a constant supportive measure, variation in MMF dosing strategies and background therapies complicates direct comparisons. Trials using monotherapy (e.g., Tang, Frisch, Maes) better isolate MMF’s effect, while newer trials incorporating systemic corticosteroid co-administration may represent more clinically viable approaches moving forward [47,51].

### 3.3. Efficacy of MMF Treatment in IgAN

Outcomes assessed include reductions in proteinuria, the stabilization of serum creatinine, the preservation of eGFR, and progression to ESΚD. Table 3 provides a structured overview of key efficacy outcomes across pivotal studies.

The overall findings are mixed. Early evidence from Tang et al. (2005) demonstrated an 80% reduction in proteinuria in MMF-treated patients, compared to 30% in controls [50]. This was complemented by long-term follow-up data from Tang et al. (2010), which showed that only 10% of patients in the MMF arm progressed to ESRD over six years, compared to 45% in the control group [46]. These data, derived from Chinese cohorts, strongly support MMF’s protective effect when initiated early and in patients with active disease.

In contrast, several Western studies failed to demonstrate similar benefits. The study from Hogg et al., a rigorously designed North American multicenter RCT, showed no significant difference in urinary protein-to-creatinine ratio (UPCR) or remission rates between MMF and placebo groups [52]. Similarly, trials by Frisch et al. and Maes et al. found no improvement in renal function or delay in ESKD progression [48,49]. These null results have fueled ongoing debate regarding MMF’s generalizability beyond East Asian populations.

As already described, Hou et al. (2017) investigated MMF in combination with low-dose corticosteroids compared to full-dose corticosteroids and found comparable efficacy in proteinuria remission, but with fewer adverse events in the MMF arm [47]. This highlights MMF’s potential role as a steroid-sparing agent with a superior safety profile in selected patients.

**Figure 1:**
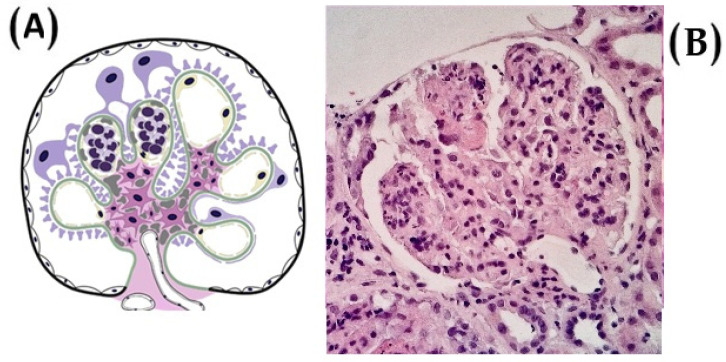
(**A**) An illustration of endocapillary hypercellularity, commonly referred to as the ‘E’ lesion, a histopathological feature associated with a worse clinical course in patients who do not receive immunosuppressive therapy. (**B**) The presence of endocapillary hypercellularity in even a single glomerulus is sufficient to classify the biopsy as E1 (image from light microscopy with hematoxylin and eosin staining, ×400).

More recently, Hou et al. (2023) conducted a large-scale RCT in China that reaffirmed MMF’s clinical efficacy [51]. The trial reported a hazard ratio of 0.23 for the primary composite endpoint (defined as sustained decline in kidney function), confirming MMF’s value in delaying disease progression. Importantly, as already mentioned, this study included histologic stratification, with many patients exhibiting endocapillary hypercellularity (E1) and crescent formation (C1/C2), lesions known to be associated with active glomerular inflammation and greater response to immunosuppressive therapy [53,54,55]. Endocapillary hypercellularity is depicted in Figure 1.

### 3.4. Histologic Stratification and Predictive Markers of MMF Response

Recent evidence underscores the pivotal role of histopathologic features, particularly indicators of intraglomerular inflammation, in predicting therapeutic response to MMF in IgAN. Among these, endocapillary hypercellularity and the presence of crescent formation, reflected in the “E” and “C” components of the Oxford MEST-C classification, have emerged as key markers of immunologic activity that may confer responsiveness to immunosuppressive interventions.

Table 4 summarizes how histological stratification was applied across selected clinical trials evaluating MMF, and in what manner this influenced study outcomes. A growing body of evidence suggests that the efficacy of immunosuppressive therapies may depend not only on disease stage and kidney function but also on the active versus chronic nature of underlying glomerular injury.

In Hou et al. (2017), histologic stratification was directly implemented, with inclusion criteria focusing on patients with active lesions, specifically crescents and endocapillary proliferation [47]. The trial demonstrated favorable clinical responses in the MMF plus low-dose corticosteroid group, with similar remission rates but a significantly improved safety profile [47]. This supports the hypothesis that immunosuppression yields the most benefit in the context of ongoing intraglomerular inflammatory damage, rather than in chronic sclerotic disease.

Tang et al. (2005) also selected patients based on mild to moderate histologic lesions [50]. While less detailed than later studies in terms of specific scoring systems, the approach nonetheless focused on patients with reversible structural damage. The trial found significant reductions in proteinuria in the MMF group, reinforcing the concept that disease activity and histologic acuity are important determinants of treatment responsiveness.

Hou et al. (2023) used CKD stages 2–3 as a clinical surrogate for histologic status, ensuring that participants had adequate renal reserve and were likely to harbor active, modifiable disease [51]. Although this approach did not rely on Oxford MEST classification, the stratification was sufficient to yield clear therapeutic benefit, with MMF significantly reducing progression to adverse renal outcomes.

Conversely, Hogg et al. and Frisch et al. did not incorporate histologic stratification in trial design [49,52]. Patients in these Western cohorts were enrolled irrespective of lesion activity, with Frisch et al. including individuals with advanced fibrosis and sclerosis, histologic features known to correlate poorly with immunosuppressive response [49,53]. Predictably, these trials failed to demonstrate clinical benefit from MMF, highlighting the critical importance of targeted patient selection.

**Figure 2:**
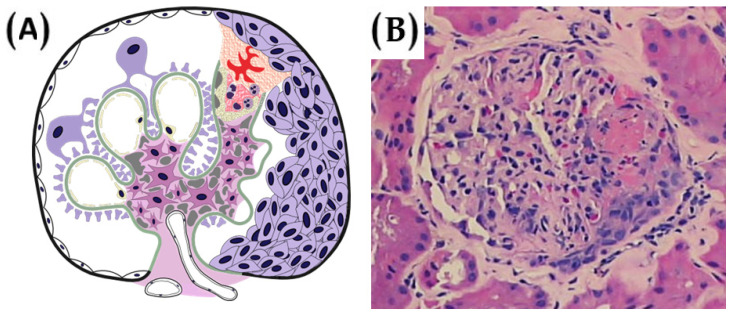
(**A**) A depiction of crescentic lesions, classified as ‘C1′ or ‘C2′ under the Oxford MEST-C scoring system. C1 lesions, defined by the presence of crescents in even a single glomerulus, are generally associated with a favorable response to immunosuppressive therapy. In contrast, C2 lesions, characterized by crescents in 25% or more of glomeruli, are typically linked to poor renal prognosis, irrespective of the immunosuppressive regimen administered. (**B**) The presence of crescents does not necessarily indicate rapidly progressive glomerulonephritis in the absence of rapidly worsening kidney function. Rather, it primarily reflects active glomerular inflammation.

Taken together, these findings illustrate that active histologic lesions such as endocapillary hypercellularity and cellular crescents are associated with more favorable outcomes following MMF therapy. In contrast, trials enrolling patients with predominantly chronic lesions or without histologic stratification generally reported no therapeutic advantage. Future trials should universally adopt standardized scoring systems, such as the Oxford MEST-C classification. Incorporating histologic criteria into trial design is essential for improving both the precision and efficacy of MMF-based therapies in IgAN. Crescents are depicted Figure 2.

## 4. Discussion

Collectively, the seven clinical studies reviewed present a detailed and, at times, conflicting picture regarding the role of MMF in the treatment of IgAN. While the most compelling evidence in favor of MMF originates from East Asian populations, particularly China, Western studies have generally failed to confirm these benefits. This divergence may be attributed to several factors, including differences in patient selection, disease chronicity, histopathologic activity, genetic background, and study design. Trials from China, such as those conducted by Hou et al. and Tang et al., demonstrated significant reductions in proteinuria and a lower risk of progression to ESKD [46,47,50,51]. These findings were derived from large-scale, well-structured randomized controlled trials that incorporated histologic stratification, adequate follow-up, and standardized co-administered therapies. In contrast, Western trials, including those by Hogg, Frisch, and Maes, were smaller, methodologically less rigorous in terms of patient stratification, and failed to show statistically significant benefits [48,49,52]. The failure to account for active histological lesions, such as endocapillary hypercellularity or crescent formation, likely diluted any therapeutic effects that MMF might have conferred.

### 4.1. MMF Dosage

Based on the available evidence from the studies analyzed, the commonly used MMF regimen for IgA nephropathy begins with 500 mg twice daily, with dose escalation to 1.000 mg twice daily after several weeks if tolerated. The initial treatment phase typically lasts 4–6 months, with discontinuation advised in cases of worsening eGFR or persistent/progressive proteinuria. For patients who continue therapy beyond this period, MMF is generally maintained for a total duration of 12 months, followed by gradual tapering, either 250 mg every two weeks or 500 mg monthly, to minimize relapse risk and adverse effects. In cases of inadequate response or treatment failure with MMF in combination with corticosteroids, therapeutic strategies are evolving rapidly. Several novel agents have recently emerged, with targeted-release budesonide, sparsentan, and iptacopan already receiving FDA approval for IgA nephropathy. Furthermore, a number of international clinical trials are currently underway, and their results are expected to further expand the range of evidence-based therapeutic options in the near future.

### 4.2. Randomized Controlled Trials

RCTs conducted in China were large-scale, well structured, and incorporated histologic stratification, adequate follow-up, and standardized co-administered therapies. In contrast, Western trials, including those by Hogg, Frisch, and Maes, were smaller, lacked histologic stratification, and failed to show statistically significant benefits [48,49,52].

Histologic activity appears to be a pivotal determinant of therapeutic response. In Hou et al. (2017), which included patients with endocapillary proliferation and crescents, MMF combined with low-dose corticosteroids achieved comparable remission rates to standard-dose corticosteroids but with a significantly better safety profile [47].

### 4.3. Observational Studies

Two observational studies (both published by Tang et al.) provide additional insights into MMF use in IgAN. Tang et al. (2010) followed the original RCT cohort for six years, reporting markedly lower ESKD incidence in the MMF group versus controls (10% vs. 45%) [46]. While these findings suggest durable benefit, the lack of randomization, potential loss to follow-up, and changes in co-interventions during the observation period introduce confounding factors that limit causal inference. Nevertheless, such extended follow-up is rare in IgAN research and offers valuable context for long-term MMF effects.

### 4.4. Disease Activity and Chronicity

Histologic activity, in particular, appears to be a pivotal determinant of therapeutic response. Trials that selected patients with active proliferative lesions (e.g., E1 or C1/C2 lesions based on the Oxford MEST-C classification) consistently demonstrated more favorable outcomes. In the study by Hou et al. (2017), which included patients with endocapillary proliferation and crescents, MMF combined with low-dose corticosteroids led to similar efficacy in terms of remission rates compared to standard-dose corticosteroids, but with a significantly better safety profile [47]. Similarly, Tang et al. (2005) enrolled patients with mild to moderate histological lesions and observed a substantial reduction in proteinuria [50]. On the other hand, studies that either failed to implement histologic stratification or included patients with extensive chronic lesions, such as glomerulosclerosis or tubulointerstitial fibrosis, were more likely to report null results. For example, Hogg et al. (2015) did not use the Oxford classification or any histologic selection criteria, and Frisch et al. (2005) included patients with advanced sclerosis, leading to a lack of demonstrable benefit [49,52].

Another key source of variation among the trials was the duration of treatment and follow-up. Studies with short durations (e.g., 6–12 months) may not adequately capture the delayed but clinically meaningful benefits of immunosuppressive therapy in a slowly progressive disease such as IgAN. The 6-year observational follow-up by Tang et al. (2010) offered rare long-term insights, revealing a lower incidence of ESKD in MMF-treated patients compared to controls [46]. Similarly, the large-scale trial by Hou et al. (2023), which followed patients for three years, demonstrated a hazard ratio of 0.23 for the primary renal endpoint in the MMF group, one of the most compelling data points in support of MMF efficacy [51]. In contrast, shorter and underpowered studies with limited observation windows were less likely to detect therapeutic effects, especially when hard outcomes such as eGFR decline or time to ESKD were not measured.

Population differences are another critical factor that may underlie the disparate findings. East Asian populations may exhibit distinct immunologic and genetic profiles compared to Western cohorts. Several factors may explain why Chinese patients appear to respond more favorably to treatment. Higher circulating levels of galactose-deficient IgA1, a greater prevalence of active histologic lesions, and potentially distinct pharmacokinetics or metabolism of immunosuppressive agents such as MMF may all contribute to the observed therapeutic efficacy in this population. These observations support the notion that MMF’s efficacy is not universally translatable and highlight the need for geographically and biologically stratified treatment paradigms.

Furthermore, background therapy, including renin–angiotensin system (RAS) blockade and the co-administration of corticosteroids or dietary interventions, varied among studies and may have introduced confounding effects. While RAS blockade was uniformly applied across all trials, the additional use of omega-3 fatty acids sodium restriction, or concurrent corticosteroids may have interacted with MMF to produce differential effects [47,48,52]. It remains unclear whether MMF’s efficacy is primarily as monotherapy or in synergy with low-dose steroids; however, the latter appears promising, especially in reducing steroid-associated adverse events.

Sample size and statistical power also played a critical role in shaping the outcomes of these trials. The largest and most recent studies by Hou et al. enrolled over 170 patients and were powered to detect clinically relevant differences. In contrast, earlier Western studies enrolled 30–50 participants, making them vulnerable to statistical error. These limitations underscore the importance of adequately powered, biopsy-driven, and stratified trials in defining MMF’s place in the therapeutic algorithm of IgAN.

Taken together, these findings suggest that MMF is not universally effective for all IgAN patients but may have significant benefit in carefully selected subgroups [29]. Specifically, patients with early-stage disease, preserved renal function, persistent proteinuria despite supportive care, and active histologic lesions appear to derive the most benefit. These characteristics should inform clinical decision-making and guide the selection of patients for immunosuppressive therapy. Importantly, the current KDIGO 2021 guidelines recommend the initiation of immunosuppressive therapy in patients with proteinuria exceeding 1 g/day despite at least three months of optimized supportive care, provided that the estimated glomerular filtration rate (eGFR) remains above 30 mL/min/1.73 m^2^ [10]. However, these recommendations do not mandate histologic stratification, a gap that recent evidence strongly suggests should be addressed.

It is worth noting that no study in the current literature has investigated the co-administration of MMF with TRB in IgAN. While the combination of TRB with systemic corticosteroids has been proposed in the literature, the concurrent use of MMF with budesonide could also represent a promising alternative [2]. This approach may serve to reduce the need for systemic corticosteroids and their associated adverse effects. Given that patients with active immunologically mediated intraglomerular inflammation appear to respond favorably to MMF, the combination with budesonide, which acts early in the immunological cascade by reducing the production of galactose-deficient IgA1, could represent a viable and effective therapeutic option.

### 4.5. Limitations

Several limitations should be acknowledged when interpreting the findings of this review. First, many of the included studies were limited by small sample sizes and inadequate statistical power, particularly in the Western sub-populations, increasing the risk of statistical error. Secondly, the follow-up duration in several trials was relatively short compared to the slow progression of IgAN, potentially underestimating long-term therapeutic effects or adverse outcomes. Third, heterogeneity in trial design, including differences in MMF dosing regimens, background therapies, and patient selection criteria, complicates direct comparisons between studies. Notably, some trials did not incorporate histologic stratification, which recent evidence suggests is critical for identifying patients most likely to benefit from immunosuppressive therapy. Furthermore, the majority of high-quality data are derived from East Asian populations, and their applicability to other ethnic groups remains uncertain. Finally, gaps in the literature persist regarding MMF in combination with novel agents or alternative immunosuppressive regimens, which should be addressed in future large-scale, biopsy-driven, and ethnically diverse randomized controlled trials.

## 5. Conclusions

IgAN remains a complex and heterogeneous glomerular disease with highly variable clinical trajectories. While immunosuppressive therapy, particularly with MMF, has shown therapeutic promise, especially in patients with early-stage disease and histologically active lesions, its wide application across all IgAN phenotypes is not supported by the current body of evidence. The therapeutic utility of MMF appears most pronounced in individuals demonstrating endocapillary hypercellularity and cellular crescents, histologic features indicative of active immune-mediated glomerular injury. In contrast, patients with advanced chronic changes or minimal histologic activity derive limited benefit.

In this context, MMF, administered either as monotherapy or in combination with low-dose corticosteroids, may serve as a viable and safer alternative to high-dose systemic corticosteroids. This steroid-sparing strategy holds particular relevance for patients with contraindications to corticosteroid therapy or increased susceptibility to steroid-associated complications. Additionally, MMF presents a more accessible and cost-effective option compared to novel targeted therapies, which, despite recent FDA approval, may remain unavailable or unaffordable in many healthcare settings for the foreseeable future. To refine MMF’s role in IgAN therapy, future clinical trials should incorporate histologic enrichment strategies, extend treatment and observation durations, and evaluate MMF in combination regimens. Moreover, the adoption of standardized histologic classifications, such as the Oxford MEST-C score, is critical to stratify patients based on disease activity and chronicity. The integration of biopsy data into personalized treatment algorithms will enhance therapeutic precision and maximize clinical benefit. Trials should also explore the development and validation of non-invasive biomarkers to predict immunologic responsiveness, allowing for better patient selection.

Until such data become available, clinical decision-making must remain individualized. MMF should be considered in Chinese patients who meet specific clinical and histopathological criteria: biopsy-proven active proliferative lesions, moderate-to-high proteinuria refractory to optimized supportive care, and preserved renal function. In these cases, MMF offers a reasonable balance of efficacy, safety, and accessibility. Personalized treatment, guided by histologic findings, clinical indicators, and geographic considerations, represents the most rational strategy for integrating MMF into the evolving therapeutic landscape of IgA nephropathy.

## Figures and Tables

**Table 1:** Summary of clinical trials evaluating mycophenolate mofetil (MMF) in IgA nephropathy: design characteristics, study populations, and treatment duration.

Study	Location	Sample Size	Design	Intervention	Duration
Tang et al., 2005 [50]	China	40	RCT	MMF vs. control	24-week + 72-week follow-up
Tang et al., 2010 [46]	China	40	Observational	MMF vs. control	6-year follow-up
Hogg et al., 2015 [52]	USA/Canada	52	RCT	MMF vs. placebo	12 months
Frisch et al., 2005 [49]	USA	32	RCT	MMF vs. placebo	24 months
Maes et al., 2004 [48]	Belgium	34	RCT	MMF vs. placebo	36 months
Hou et al., 2017 [47]	China	176	RCT	MMF + low-dose steroid vs. steroid	12 months
Hou et al., 2023 [51]	China	170	RCT	MMF + SC vs. SC	36 months

**Table 2:** MMF dosing, co-administered therapies, and RAS blockade across key clinical trials in IgA nephropathy.

Study	MMF Dose	Co-Administered Therapy
Tang et al., 2005 [50]	1.5–2 g/day	RAS blockade
Tang et al., 2010 [46]	Uknown	RAS blockade
Hogg et al., 2015 [52]	25–36 mg/kg/day	ACEi/ARB + omega-3 fatty acids
Frisch et al., 2005 [49]	1 g twice daily (BID)	ACEi/ARB
Maes et al., 2004 [48]	2 g/day	ACEi + sodium restriction
Hou et al., 2017 [47]	1.5 g/day	RAS blockade + low-dose corticosteroids
Hou et al., 2023 [51]	1.5 g/day tapered to 0.75–1 g/day	Losartan

**Table 3:** Clinical efficacy outcomes of MMF in IgAN across major clinical trials.

Study	Outcome	Conclusion
Tang et al., 2005 [50]	80% reduction in proteinuria vs. 30% in control	Significant benefit
Tang et al., 2010 [46]	10% ESRD in MMF vs. 45% in control (6-year data)	Long-term protection
Hogg et al., 2015 [52]	No significant difference in UPCR or remission	No benefit
Frisch et al., 2005 [49]	No difference in serum Cr or ESRD	No benefit
Maes et al., 2004 [48]	No significant change in renal outcomes	No benefit
Hou et al., 2017 [47]	Similar remission rates; fewer adverse effects with MMF	Safer profile
Hou et al., 2023 [51]	HR of 0.23 for primary renal endpoint (favoring MMF)	Significant benefit

**Table 4:** Histologic stratification and predictive markers for MMF response in IgA nephropathy.

Study	Histological Criteria	Stratification Used	Interpretation
Hou et al., 2017 [47]	Crescents, endocapillary hypercellularity	Yes	Favorable response
Tang et al., 2005 [50]	Mild to moderate lesions	Yes	Improved proteinuria
Hou et al., 2023 [51]	CKD stage 2–3 (clinical surrogate)	Yes	Reduced progression
Hogg et al., 2015 [52]	No histologic stratification (Oxford MEST absent)	No	No benefit
Frisch et al., 2005 [49]	Advanced fibrosis, sclerosis	No	No benefit
